# Localized soft elasticity in liquid crystal elastomers

**DOI:** 10.1038/ncomms10781

**Published:** 2016-02-23

**Authors:** Taylor H. Ware, John S. Biggins, Andreas F. Shick, Mark Warner, Timothy J. White

**Affiliations:** 1 Materials and Manufacturing Directorate, Air Force Research Laboratory, Wright-Patterson Air Force Base, Ohio 45433, USA; 2 Department of Bioengineering, The University of Texas at Dallas, Richardson, Texas 75080, USA; 3 Cavendish Laboratory, Cambridge University, Cambridge CH3 0HE, UK

## Abstract

Synthetic approaches to prepare designer materials that localize deformation, by combining rigidity and compliance in a single material, have been widely sought. Bottom-up approaches, such as the self-organization of liquid crystals, offer potential advantages over top–down patterning methods such as photolithographic control of crosslink density, relating to the ease of preparation and fidelity of resolution. Here, we report on the directed self-assembly of materials with spatial and hierarchical variation in mechanical anisotropy. The highly nonlinear mechanical properties of the liquid crystalline elastomers examined here enables strain to be locally reduced >15-fold without introducing compositional variation or other heterogeneities. Each domain (⩾0.01 mm^2^) exhibits anisotropic nonlinear response to load based on the alignment of the molecular orientation with the loading axis. Accordingly, we design monoliths that localize deformation in uniaxial and biaxial tension, shear, bending and crack propagation, and subsequently demonstrate substrates for globally deformable yet locally stiff electronics.

## Abstract (web summary)

Ruggedized stretchable electronic devices motivate the development of globally stretchable yet locally stiff materials. Here, Ware *et al*. programme the self-organization of liquid crystal elastomers to yield stretchable materials of homogenous composition but with spatial variation in mechanical properties.

## Graphical abstract

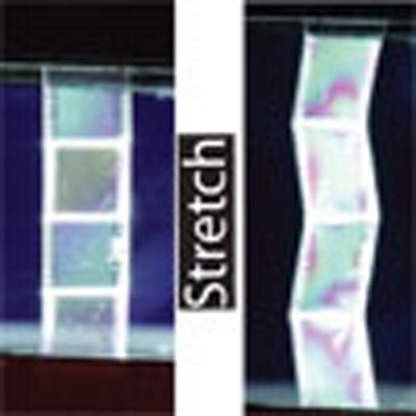

Synthesizing materials or material systems (composites) with programmable variation of mechanical properties such as stiffness, stretchability or strength coupled to functional utility such as actuation, damping or optical activity yield multifunctional materials designed *a priori* for both structural and functional utility. In natural systems, such as the interface of bone to tendon, material structure and compositions are programmed to improve or extend function^1^. A number of strategies have been explored to enable this control in synthetic materials. A considerable majority of prior efforts have employed top–down methods to locally programme stiffness via material heterogeneity^2^,^3^,^4^. The assimilation of functional performance into a monolithic device of a single composition would reduce or remove the need to add functional mechanisms and their accompanying assemblies and diminish the need for structural inclusions to increase or decrease stiffness. One of the first steps towards the preparation of functional material systems are hybrid devices, such as flexible electronics. In the recent literature a number of approaches for preparation of ruggedized flexible devices have been reported primarily focusing on the general strategy of preparing elastomeric composites with programmed mechanics. The primary method of programming variation (spatial heterogeneity) in the mechanical response of these flexible and stretchable composites has been to vary either composition or structure. For example, magnetically oriented anisotropic particles have been shown to control tensile, wear and shear properties^5^,^6^,^7^. Further, top–down methodologies such as photolithography have been employed to pattern crosslink density or islands of high-performance polymers within an elastomeric matrix to tune both the local and global response to an applied load^2^,^8^. These advances have been accompanied by demonstrated utility in realizing globally compliant and locally stiff stretchable electronics. Anisotropy generated by localizing the self-assembly of materials such as liquid crystal polymer networks (LCNs) or elastomers (LCEs) could offer potential benefits afforded by improvements in resolution and ease of fabrication.

LCNs and LCEs have been widely studied in part due to the correlation of the molecular orientation to mechanical properties and ultimately to a stimulus response. The distinguishing feature of LCNs from LCEs is crosslink density, which accordingly affects the glass transition temperature, the magnitude of the order induced changes when subjected to a stimulus, and the response to a mechanical load as detailed in a recent review^9^. Glassy LCN have been shown to have anisotropic mechanical properties but tend to fail at very small strains (>3–10%) limiting the utility of these films for applications that require large deformation to load^9^. By comparison, LCEs are capable of large, anisotropic and nonlinear deformations similar to that of many biological materials^10^. When an LCE is deformed along the alignment direction (nematic director) the materials exhibit a classical elastic response. However, if the material is deformed orthogonally to the alignment direction (nematic director) a comparatively ‘soft' elastic response has been reported, in which the material stretches at near zero stress as it simply adopts another equivalent state with a different alignment. This phenomenon in LCEs is commonly referred to as ‘soft elasticity' and attributed to the reorientation of the nematic director of the LCE to align along the direction of the stretch. The ability to localize or pattern the director within LCEs could yield strain-patterning in chemically homogenous monoliths^11^.

LCEs have historically been prepared by methods employing mechanical or magnetic alignment^12^,^13^,^14^. The complex alignment of LCEs by mechanical or magnetic fields is inherently limited in spatial resolution, and nearly all prior examinations of soft elasticity have been undertaken in samples prepared with uniaxial alignment of the director. In recent years, optical alignment of surface layers has been used to spatially control the local orientation of LCNs (refs 15, 16). The resulting monoliths have been examined for their potential utility as complex sensors and actuators. Recently a synthetic method and processing technique was reported to prepare arbitrarily patterned LCEs, using photoalignment of a photochromic surface coating and the ability of certain formulations of liquid crystalline monomers to self-assemble to these treated surfaces^17^. In the work presented here, we use this methodology to functionally grade the order of LCEs to design the global mechanical properties of a polymeric sample of homogenous composition. Leveraging the distinctive soft elastic properties of the materials, we demonstrate that self-assembly of LCEs can yield spatially localized mechanical responses in tension, biaxial tension, bending and crack management. Programmed monolithic substrates of homogenous composition are prepared that are globally stretchable and locally stiff elucidating a new strategy to yield designer substrates for flexible devices.

## Results

### Patterning of heterogeneous monoliths

**Figure 1:**
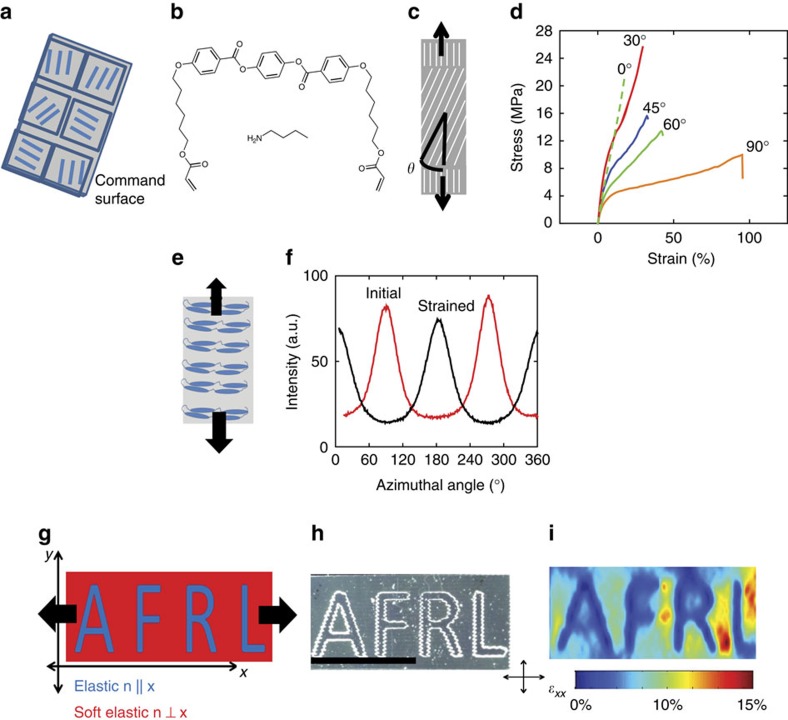
Patterned order in liquid crystal elastomers functionally grades mechanical properties. A photoaligned command surface (**a**) is used to spatially control the orientation of a main-chain LCE (**b**). Global mechanical properties are highly sensitive to the orientation of the nematic director (**c**) with respect to the loading axis (**d**). On loading a liquid crystal elastomer perpendicular to the nematic director (**e**), reorientation is observed. This reorientation has been confirmed with WAXS (**f**). By spatially patterning the order of an LCE the local strain environment can be controlled. Using point-by-point alignment, arbitrary anisotropic modulus profiles can be written (**g**,**h**). On loading, this complex monolith uniaxially strain is localized to soft elastic regions, as measured by 2D differential image correlation (**i**). Crossed arrows indicate the orientation of the polarizer and analyser for photographs. Scale bar, 5 mm.

The LCEs examined here were prepared within alignment cells with control surfaces patterned through point-by-point photoalignment of an azobenzene dye that is locally oriented at the molecular level by exposure to focused linearly polarized blue light (Fig. 1a). These ordered polymers, which self-assemble according to the patterns blueprinted into the command surfaces, are synthesized through a two-step synthetic procedure. Briefly, the monomer precursors are filled into the patterned alignment cell. The cell consists of two pieces of glass each coated with the molecularly aligned azobenzene dye. These pieces of glass are glued together with a predefined gap between the inner surfaces of 50 μm. After filling, a slow chain-extension reaction proceeds to yield end-capped diacrylate oligomers. On completion of the chain-extension reaction, the materials are subsequently photopolymerized (Fig. 1b) yielding a lightly crosslinked elastic solid where the order is permanently trapped. The order parameter of this aligned material is near 0.5 (ref. 16). This reaction scheme enables complex alignment of the nematic director to produce LCEs with main-chain mesogens. Within an area of uniform alignment, the polymer can be considered to be transversely isotropic, as the nematic order is uniaxial, analogous to fiber-reinforced polymer composites or many biological connective tissues^18^,^19^. Along the alignment direction (nematic director), the properties of the polymer such as modulus, strain-to-failure and coefficient of thermal expansion are distinct from the orthogonal axes. Depending on the polarization of the light used to pattern the command surface the director can be varied within a film. Under load, the director orientation with respect to the loading axis controls the global stress–strain response of the film (Fig. 1c). The stress–train behaviour of uniformly aligned LCEs prepared with this method are presented in Fig. 1c in which the alignment of the nematic director is varied from parallel (0°) to intermediate (30°, 45°, 60°) as well as perpendicular (90°) to the gauge length. For orientations of the director that are not aligned with the load, a highly nonlinear stress–strain response is observed. After the initial linear behaviour, a semi-soft plateau is observed. The length of this plateau increases while the slope of the plateau decreases, as the initial director is increasingly misaligned from the loading axis. It should be noted that in addition to stress along the principal axes, the contribution of in-plane shear can also be observed, as discussed below. The variations in the shapes of the semi-soft plateaus evident in Fig. 1d can be attributed to director reorientation. An azimuthal scan of wide angle X-ray scattering confirms the expected 90° rotation of the director at a strain of 120%, just before failure, is shown in Fig. 1e,f. Utilizing the ability to spatially control the orientation of the director it was hypothesized that under uniaxial tension, strain could be localized to regions where the director is misaligned from the loading axis.

### Designing local mechanical response

A schematic of a film with an arbitrary domain pattern is shown in Fig. 1g,h. The soft elastic (90°) regimes are aligned perpendicularly to the load while the elastic domains (0°) are aligned along the load axis. After fabrication, the pattern is visible between crossed polarizers. Both elastic and soft elastic domains are optically equivalent and dark between crossed polarizers, but a bright boundary between the two domains is visible. Using two-dimensional (2D) digital image correlation (DIC), the strain on the sample under tension can be mapped (Fig. 1i). The strain evident in the patterned domains differs by a factor of 5 (referred to hereafter as strain contrast ratio) with the soft elastic regions deforming and the elastic regions remaining relatively unchanged (Supplementary Fig. 1). To design a functional and spatially patterned elastomeric substrate two factors must be controlled, the macroscopic orientation of the sample (that is, what fraction of the elastomer domains are oriented in which direction) and the geometry of the sample (that is, how are the domains arranged spatially). Here we focus on the limit where the continuous domain are misaligned with the loading axis to enable spatial control of strain.

**Figure 2:**
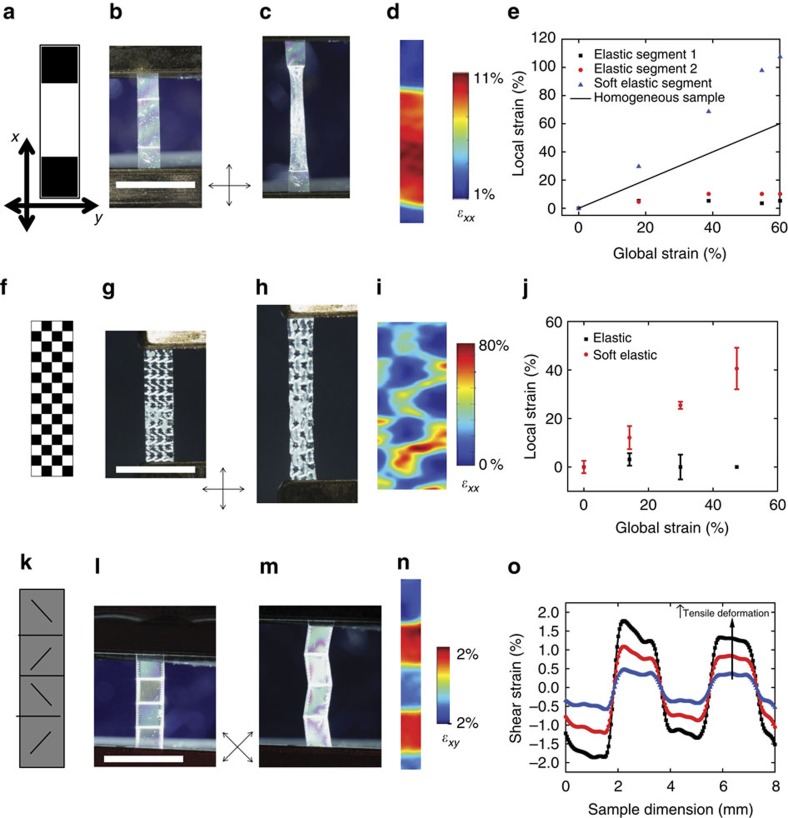
Both extensional and shear strain can be controlled in 2D. The desired pattern of modulus is indicated in **a**,**f** and **k** when loaded along the long axis of the film. Black pixels correspond to elastic regions while white pixels correspond to soft elastic regions. Grey scale values are indicative of angle of the director. Images of the fabricated LCE film with the desired director pattern (**b**,**g**,**l**) and after straining (**c**,**h**,**m**) between crossed polarizers. 2D DIC is used to map the strain of the sample (**d**,**i**,**n**) at 12% global tensile strain. For patterns with only elastic and soft elastic regions (**d**,**i**) extensional strain is shown. Shear strain is shown for *n*. Strain is plotted on the undeformed sample grid. The local extensional (**e,j**) and shear (**o**) strains for these heterogeneous films are shown for distinct director orientations as a function of global extensional strain. Error bars (**j**) indicate s.d. of *n*=5 regions of the same director orientation. Shear strains (**o**) are shown along the sample lengths at 4, 8 and 12% global tensile strain. Crossed arrows indicate the orientation of the polarizer and analyser for photographs. Scale bars, 6 mm.

Binary control of the director orientation (along or perpendicular to the loading axis) can be used to spatially control uniaxial tensile strain. While the modulus differs by a factor of 6 in the linear elastic regime of the stress–strain curve, the stress plateau can greatly increase the contrast in strains at large deformations in the soft elastic regime. Figure 2a illustrates this concept in a sample prepared with three domains, two elastic domains at the extremes and one soft elastic domain in the central region of the LCE. On deformation, the strain is largely contained in the central soft elastic region (Fig. 2b,c). Using DIC, a strain contrast ratio of approximately 5 is observed at 10% strain (Fig. 2d). On further deformation the nonlinear behaviour leads to a strain contrast ratio of greater than 15 at 60% global strain (Fig. 2e). This corresponds to greater than100% strain in the soft elastic domain where reorientation of the director is observed. This highly nonlinear behaviour is characteristic of LCEs exhibiting soft elasticity. Soft elastic domains can also be patterned on a much smaller scale. Due to the nature of the optically directed self-assembly technique used to fabricate these LCEs, the localization of strain is not limited to relatively large domains (∼10 mm^2^). Demonstrating this ability, a pattern of 60, 0.25 mm^2^ (Fig. 2f) square domains were patterned into a single film. The soft elastic domains preferentially deform as evident in the DIC analysis as well as optical characterization (Fig. 2g–j). It should be noted that this spatially controlled deformation also leads to spatially controlled changes in thickness due to the Poisson's effect (Supplementary Fig. 2). As shown in Fig. 1, deformation perpendicular to the director induces reorientation of the director. After releasing the load, a slow recovery is observed at room temperature. This mechanical hysteresis is dependent on the ambient temperature. For instance, recovery occurs rapidly after heating to 50 °C.

Thus far we have demonstrated control of strain along the loading axis (*ɛ*_*xx*_ ) and as well as strain orthogonal to the loading axis (*ɛ*_*yy*_ and *ɛ*_*zz*_; see also Supplementary Figs 1 and 2). As indicated by the planar strain tensor shown in equation (1), control of shear strains, *ɛ*_*xy*_, enables full control


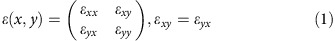


of strain in 2D. Shear strains arise in transversely isotropic materials when the loading direction is not parallel or perpendicular to the preferred axis, in this case, the liquid crystal director. Utilizing the continuous control of director orientation provided through optical patterning, we introduced domains with director orientations at ±45° to the loading axis (Fig. 2k,l). On deformation, strain is split between the principal and shear planes. The result is an off-axis deformation where the sign of the shear strain depends on the handedness of the reorientation of the director, which is controlled by the sign of the angle the director orientation (Fig. 2m) makes with the loading axis. As the different domains have the same magnitude of intermediate orientation from the loading axis, little contrast exists in tensile strain or magnitude of shear strain. However, the sign of the shear strain is opposite for the two distinct domain types (Fig. 2n,o). Control over the magnitude and directionality of shear strain in viscoelastic materials enables controllable damping and is an area of ongoing interest.

### Designing substrates for stretchable electronics

**Figure 3:**
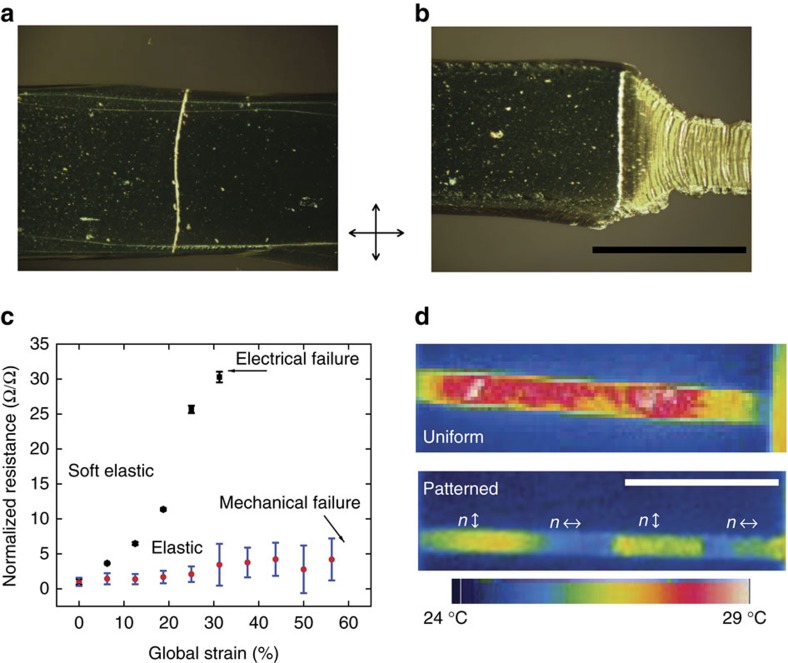
Patterned director orientation isolates strain–sensitive components for stretchable electronics. A transmission optical micrograph of the silver coated LCE before (**a**) after deformation (**b**) showing clear failure of the silver coating in the soft elastic regime. Scale bar, 1 mm. Resistance for a film of silver as a function of global strain is largely unchanged in domains where the director lies along the loading axis. Resistance increases by 30-fold in domains where the director lies perpendicular to the loading axis (**c**). Error bars indicate s.d. of *n*=3 samples. Infrared images of a uniform (top) and patterned (bottom) LCE film coated with silver nanowires, strained to 10% global strain and with an applied voltage along the long axis of the film (**d**). Patterned director orientation leads to patterned resistance in the film. Scale bar, 5 mm. Crossed arrows indicate the orientation of the polarizer and analyser for photographs.

The vast majority of materials used in conventional electronic devices fail at strains orders of magnitude lower than the failure strain of elastomers^8^. As a result, electrical components in traditional geometries fail when built on substrates that undergo large and dynamic strains. This has motivated significant recent attention focused on improving the stretchability of electronic materials as well as strategies to localize strain within compliant materials. By coating patterned LCE films with a conducting layer, such as thin film silver, a spatially variable strain evolves in the coating under load. In Fig. 3a,b, optical micrographs show the interface between soft elastic and elastic domains before and after deformation. After deformation, the soft elastic domain has deformed considerably leading to visible failure of the Ag coating. Qualitatively the coating remains unchanged in the elastic domain. To elucidate this response, the resistance was measured along the length of the domain as a function of deformation in patterned LCE films coated in Ag. In the soft elastic segment of the sample, resistance increases more than 30-fold over 30% global strain before failing electrically (Fig. 3c). Over the same amount of global strain, the resistance of the coating in the elastic segment only increases threefold, and only increases slightly further before the sample fails mechanically at 60% global strain. To further improve strain insensitivity, a film of silver nanowires was deposited to serve as a ductile conductor. On deformation the soft elastic region increases precipitously in resistance as a function of strain, as has been previously reported^20^, while the elastic domains remain largely unchanged (Supplementary Fig. 3). This order-controlled resistance can be indirectly measured using infrared imaging while passing current along the long axis of the sample (Fig. 3d). Samples with only a single-domain show relatively uniform temperature profiles across the film. In contrast, a patterned sample with alternating elastic and soft elastic domains shows a periodic temperature profile, similar to several resistors in series. Higher resistance within soft elastic domains leads to localized resistive heating, as the current remains equal for all domains.

**Figure 4:**
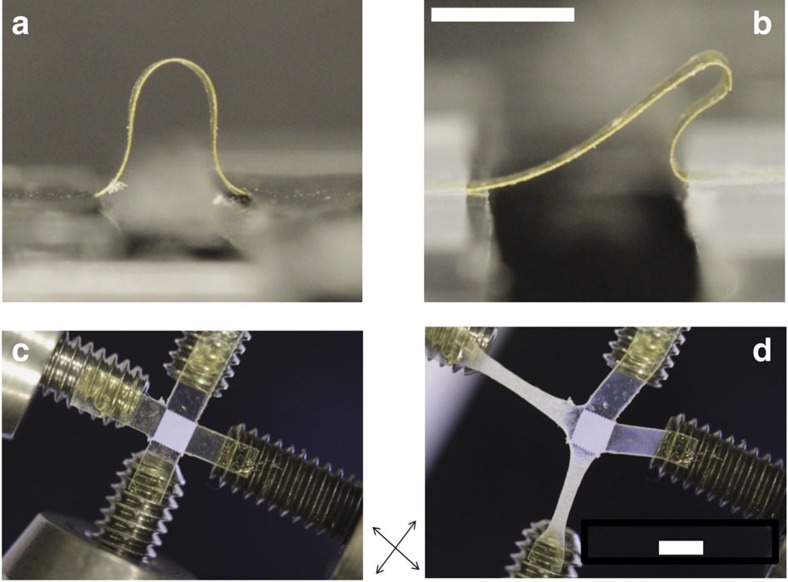
Patterned director orientation can be used to control multiaxial deformation. Buckling of a rectangular beam with uniaxial alignment (**a**) and patterned alignment (**b**) leads to the formation of symmetric and asymmetric arches, respectively. Bending is greatly reduced in areas where the director is oriented along the bending stress (along the beam long axis). Biaxially elastic behavior can be combined with local soft elasticity introduced using twisted nematic regions. A square cross with twisted nematic centre (**c**) deforms preferentially in the arms of the cross (**d**) under biaxial tension. Crossed arrows indicate the orientation of the polarizer and analyser for photographs. Scale bars, 2 mm.

Flexible and stretchable devices may not be subjected to simple uniaxial tension, but likely are required to continue functioning through a wide variety of deformations. Here we show that LCEs can be designed to localize strain in a variety of relevant loading conditions. Buckling of a doubly fixed beam leads to tensile and compressive stress along the loading axis. The bending stiffness can be readily controlled, for example by patterning a sample with two domains (Supplementary Fig. 6) with the director along and perpendicular to the long axis of the beam. On buckling, a uniformly oriented sample exhibits classic Euler buckling (Fig. 4a) while the patterned LCE exhibits a deformed arch (Fig. 4b). The domain aligned parallel to the loading axis exhibits a radius of curvature 7.5 times larger than the domain aligned perpendicularly to the load. Furthermore, this anisotropy controls deformations in complex loading environments in the plane, such as the direction of (Supplementary Fig. 4) and energy required for (Supplementary Fig. 5) crack propagation. As the local ordering of the planarly aligned nematic is only one-dimension, additional control is required to modulate response to biaxial loading environments. In fiber-reinforced composite materials, macroscopic anisotropy is often reduced by joining laminates with different orientations into a single part. Here we mimic these laminates by using the twisted nematic orientation where the director rotates by 90° through the thickness of the material at the molecular scale. In Fig. 4c,d, we fabricate and biaxially load a square cross. The intersection of the cross has a twisted nematic alignment while the arms of the cross are uniaxially aligned perpendicularly to the load directions (Supplementary Fig. 7). The result is a quasi-isotropic elastic domain that is protected from strain while the arms of the cross deform in a soft elastic manner. Previously described methods such as patterned crosslink density provide only isotropic regions of distinct modulus. Varying twist angle in aligned LCEs enables designs previously used in isotropic materials with patterned crosslink density to also be incorporated into these monolithic materials. This combination of spatial and hierarchical control of the stress–strain response of a monolith may enable a wide variety of engineered stress–strain responses on the global and local scale.

## Discussion

Here, we demonstrate the ability to locally pattern molecular orientation in LCEs as a way to functionally grade the local and global response of these flexible materials to a mechanical load. Deformations within the plane, tension, shear and crack propagation, along with out-of-plane deformations, such as buckling, can be controlled by designing the director orientation of the film. In tension, the magnitude of strain can be designed into arbitrary patterns with contrast of strain greater than 15 between elastic and soft elastic domains. Critically, this occurs within a monolith of homogenous composition and structure. We utilize these complex monoliths to design a globally stretchable conductor with locally protected domains. By directing molecular orientation in an elastomeric film, engineers now have an additional tool to programme the mechanical response of polymer substrates for mechanically sensitive devices. This technique might also be combined with patterned crosslink density or patterned reinforcement to further advance the design of heterogeneous materials.

## Methods

### Materials

1,4-Bis-[4-(6-acryloyloxyhexyloxy)benzoyloxy]-2-methylbenzene (RM82) was purchased from Synthon Chemicals (Germany). The photoalignment material, PAAD-22, was purchased as a 1% solution in dimethylformamide from BEAM Co. (USA) and was diluted to 0.33 wt% and filtered prior to use. N-butylamine was purchased from Sigma Aldrich (USA). Irgacure 369 (I-369) was provided by BASF. Silver nanowires were purchased from Seashell Technologies at a concentration of 5 mg ml^−1^ in isopropanol. Average nanowire dimensions ranged from 20 to 50 μm in length and 120 to 150 nm in diameter. All chemicals were used as received without further purification or modification unless otherwise noted.

### Optical patterning of liquid crystal cells

Photoalignment of an azobenzene dye (PAAD-22, Beam Co.) was used to pattern director orientation in liquid crystal cells. Methods used here were previously described^17^. Briefly, plasma-cleaned glass slides were coated with the photosensitive dye using spin coating from a 0.33-wt% solution in dimethylformamide. The glass was then baked for 10 min at 100 °C to remove remaining solvent. Sets of two dye coated glass slides (38 × 25 mm^2^) were then exposed to point-by-point irradiation of linearly polarized 445 nm light over an area of 0.01 mm^2^. This step is then repeated across the sample using a custom-built optical patterning setup. The dye aligns perpendicularly to the electric field vector of the incoming light. These two patterned surfaces were then spin coated with a layer of RM 257 (Merck), which is subsequently polymerized to render the alignment permanent. These two slides are then aligned and glued together using a two-part epoxy mixed with 50-μm-diameter glass spheres.

### LCE synthesis

The liquid crystal cells are then used to align a nematic mixture of monomers that can be polymerized into a LCE. A near stoichiometric mixture of RM 82 (Synthon), a nematic diacrylate and *n*-butylamine (Fig. 1b; Aldrich) with 1.5 wt% I-369 (Ciba), a radical photoinitiator, are mixed in a scintillation vial. The resulting mixture exhibits a broad nematic phase. The monomer solution is filled into the patterned liquid crystal cell using capillary action at 80 °C. This mixture is then cooled to 75 °C, allowing for alignment to the patterned surfaces and left for 16 h. During this time the mixture first undergoes a step-growth oligomerization resulting in main-chain liquid crystalline acrylate terminated macromers. The macromers are then crosslinked by exposure to 100 mW cm^−2^ ultraviolet light (365 nm) at room temperature. The resulting elastomeric film is then removed from the glass cell and cut into individual samples.

### Mechanical characterization

Mechanical characterization was performed using one of two methods. Quantitative measurement of the stress–strain response in tension was obtained using a RSA III (TA Instruments) at room temperature. Samples were rectangular and approximately 8 × 2 × 0.05 mm^3^ in size. Samples were loaded along the long axis of the film. Quantitative measurement of local strain within domains was performed optically. Films were clamped in a home-made tensile testing grip fitted with a micrometre. Strain was applied to the sample in predetermined intervals and then images of the sample were taken between crossed polarizers. Changes in domain dimensions (local strain) were obtained through image analysis. Bending measurements were performed using a home-made clamp fitted with a micrometre. All films were clamped on both ends. Images of the buckled films were taken with a digital camera and analysed in ImageJ. Curvature was measured at the centre of each domain.

### Digital image correlation

Strain mapping in 2D was performed using DIC. After synthesis, each film was spray-coated with a carbon black ink. As a result the sample was coated with a black speckle pattern that was used for image analysis (Supplementary Fig. 1). Samples were loaded into the tension grips fitted with a micrometre and strained at intervals of 3% strain where images were taken (Cannon DSLR) fitted with a macro lens. Image analysis was performed using freely distributed code run in Matlab^21^,^22^. Each image was correlated to the previously taken image. Strains were then calculated in the principal and shear directions for each image.

### Electrical characterization

Conductive coatings were applied to the LCE film by either electron beam evaporation of 50 nm of silver or by dipcoating in a solution of silver nanowires in isopropanol (Seashell Technologies). The nanowire films were then dried from solution at room temperature under gentle N_2_ flow. The coated film was then heated for 10 s at 120 °C. This process was then repeated. The resulting coatings showed a resistance of ∼15 Ω over 8 × 2 mm^2^ films. To measure the local resistance change two methods were used. The first method used direct measurement of resistance using a multimeter. Indirect measurement of resistance was made by monitoring resistive heating. Electrical contact was made using two metal clamps at the extremes of the long axis of the film. The sample was then strained to 20% global strain. Sufficient current was then applied (∼10 mA) to cause resistive heating above the ambient temperature. The spatial distribution of heat was then monitored using an infrared camera (FLIR).

## Additional information

**How to cite this article:** Ware, T. H. *et al*. Localized soft elasticity in liquid crystal elastomers. *Nat. Commun.* 7:10781 doi: 10.1038/ncomms10781 (2016).

## Supplementary Material

Supplementary Information Supplementary Figures 1-7 and Supplementary Methods
