# Supplementary material for: Localized soft elasticity in liquid crystal elastomers
Source: Nat Commun. 2016 Feb 23;7:10781. doi: 10.1038/ncomms10781 (PMC4766422; doi:10.1038/ncomms10781)
Supplement: Supplementary Information — Supplementary Figures 1-7 and Supplementary Methods [file ncomms10781-s1.pdf]

Supplementary Information

Supplementary Figures

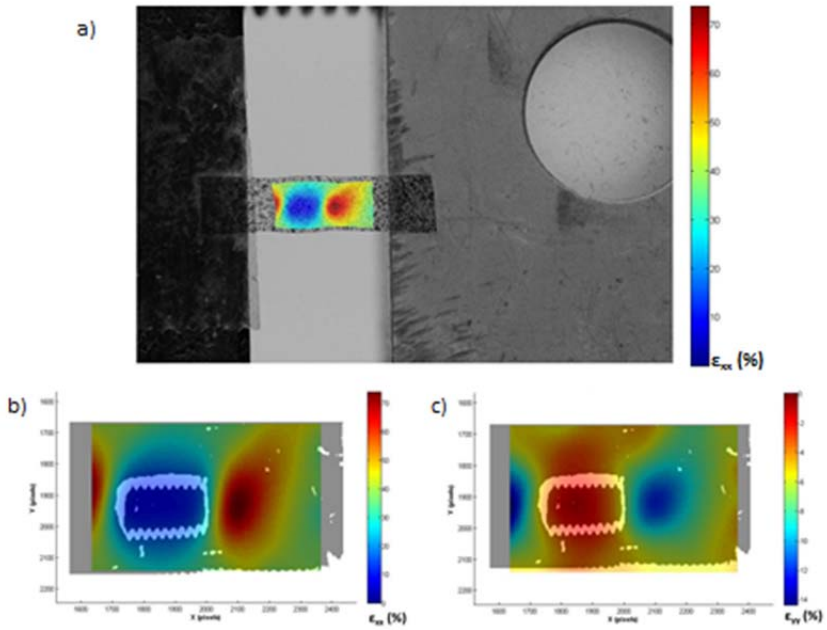

Supplementary Figure 1: Digital image correlation can be used to map strain in 2D with the patterned liquid crystal elastomers. An optical image of the patterned LCE with a speckle pattern used to measure deformation is overlayed with a contour plot of strain, ε_{xx} (A). The same contour plot of strain plotted on the original sample dimension is overlayed with an image of the patterned LCE taken between cross polarizers (B). The low strain region corresponds to the elastic domain within an soft elastic film both in ε_{xx} (B) and ε_{yy} (C).

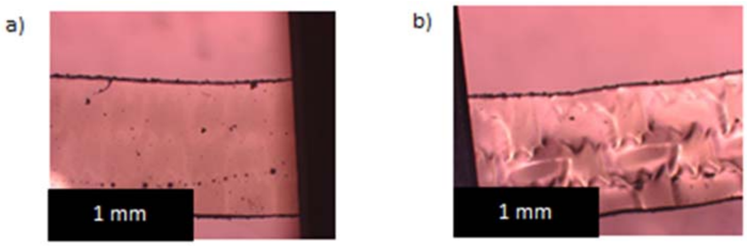

Supplementary Figure 2: Deformation of a spatially patterned LCE leads to a change in spatially controlled change in sample thickness. A sample patterned with alternating elastic and soft elastic domains in a “checkerboard” pattern (A) selectively deforms in the soft elastic domains. Due to the Poisson’s effect these domains selectively become thinner leading to a change in surface topography that can be observed optically (B).

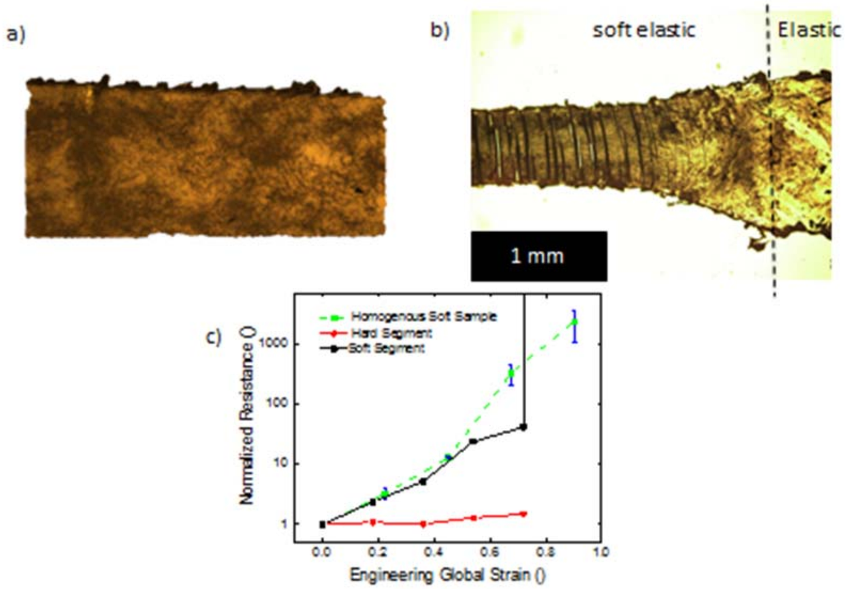

Supplementary Figure 3: Transmission optical micrographs of a patterned LCE film coated with silver nanowires before (A) and after (B) deformation. After deformation, the strain is largely contained in the soft elastic domain which leads to cracking of the nanowire film. Resistance for a film of silver nanowires as a function of global strain is largely unchanged in domains where the director lies along the loading axis. Resistance increases 3 orders of magnitude in domains where the director lies perpendicular to the loading axis (C)

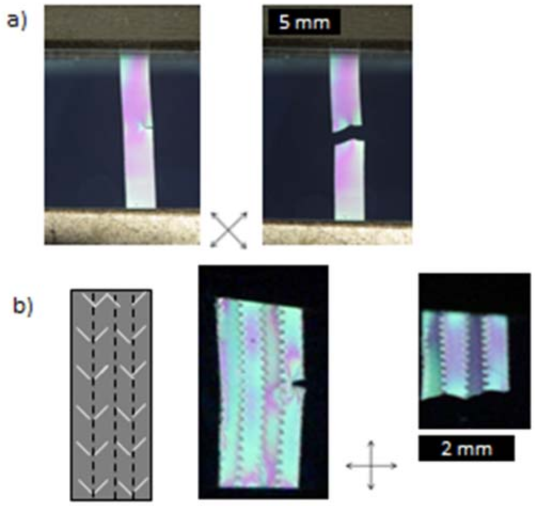

Supplementary Figure 4: Failure modes can also be controlled such as the opening of a notch. In a homogenous sample the crack proceeds in a relatively uniform manner (a). In a patterned sample consisting of alternating stripes of 45° and -45° (b) shear strain redirects the notch along the soft axis of the film.

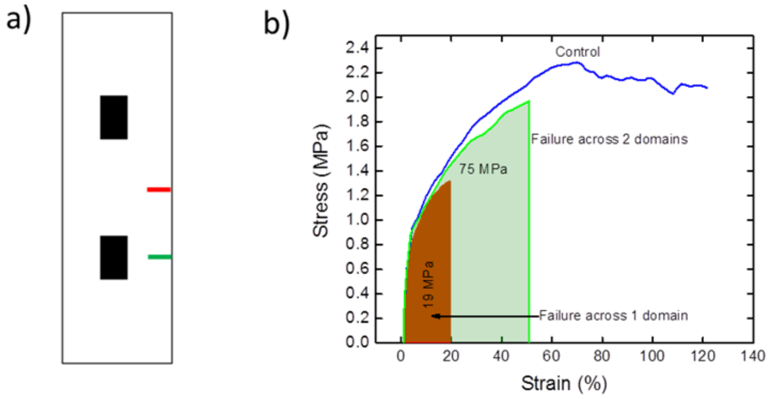

Supplementary Figure 5: Notch resistance can be introduced by placing elastic domains within a soft elastic film. When the crack path passes through the elastic domain (green crack) (a), the energy required to notch propagation is nearly 4 times higher (b) than the energy required to propagate a crack through a single domain (red crack). Crossed arrows indicate the orientation of the polarizer and analyzer for photographs.

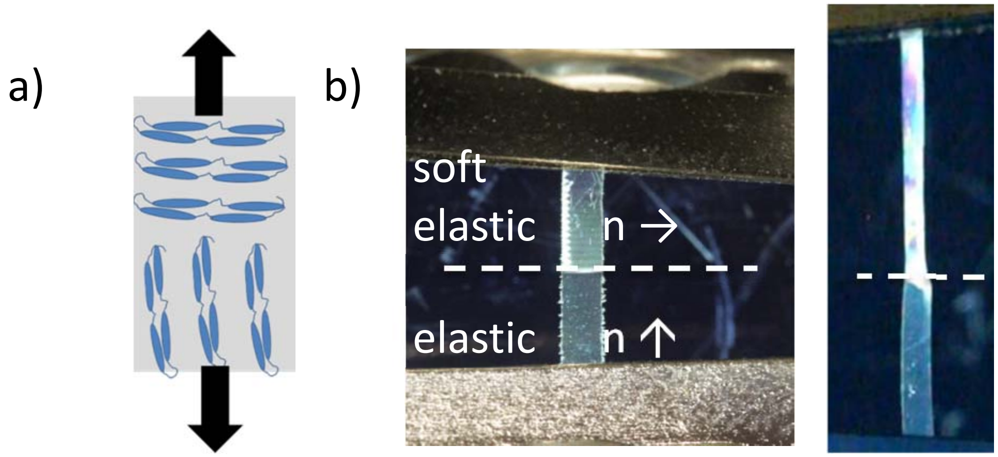

Supplementary Figure 6: A schematic of a patterned liquid crystal elastomer film with two domains, one elastic and one soft elastic. On deformation this simply patterned monolith deforms heterogeneously with strain localized in the soft elastic domain as can be observed in the photographs between crossed polarizers (B).

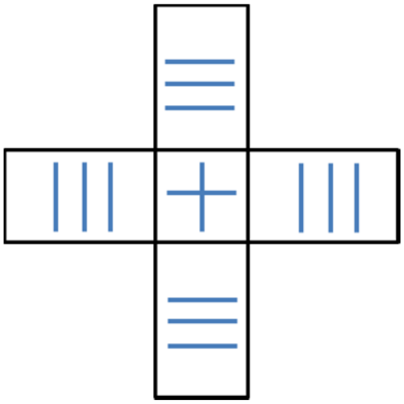

Supplementary Figure 7: Schematic of a sample geometry that exhibits elastic behavior in the twisted nematic (center domain) when loaded biaxially along the arm of each cross. Each cross arm exhibits soft elastic behavior. Director orientation is indicated by blue lines ad film outline is shown in black.

Supplementary Methods

Digital Image Correlation

Digital Image correlation was performed using freely available software run in Matlab. The results of a typical correlation are shown in **Supplementary Figure 1**. This sample consists of two domains, a soft elastic continuous domain and a rectangular elastic domain in the center of the sample. This leads to preferential deformation in the soft elastic domain. This deformation can be visualized in a variety of ways. In Supplementary Figure 1a, an overlay of the optical image of the sample under deformation and the strain in the correlated area is shown. Strains can be also plotted on the reference grid to the undeformed sample dimensions. The primary strain is plotted on the reference grid in **Supplementary Figure 1b**. This contour map is then overlaid with an image of the film before deformation under crossed polarizers. Similarly, the minor strains can be shown on the same reference grid, overlaid with the image between crossed polarizers. The area of low strain, in both axes, corresponds to the elastic domain.

It should be noted that spatially controlled magnitude of strain in the plane leads to spatially controlled changes in thickness. To demonstrate this control, optical micrographs of a patterned LCE with alternating elastic and soft elastic domains in a “checkerboard” pattern are shown in **Supplementary Figure 2**. The sample deforms from flat to alternating regions of similar height corresponding to the domains patterned into the film.

Electrical characterization

Figure S3 shows the behavior of a stretchable conductor, a film of silver nanowires, on a patterned LCE substrate. Transmission optical micrographs of the sample before (**Supplementary Figure 3a**) and after (**Supplementary Figure 3b**) deformation demonstrate the localization of strain to soft elastic domains. The nanowire coating under deformation develops cracks perpendicular to the loading axis within this soft elastic domain. This leads to electrical failure near 60% global strain. The change in resistance of the nanowire coating on elastic and soft elastic domains as a function of strain is shown in **Supplementary Figure 3c**. This behavior is compared to a homogenous sample with only a single soft domain.

Crack Opening

Type 1 crack opening was used to demonstrate anisotropic crack propagation. In a uniform sample, a monodomain aligned perpendicular to the loading axis with a sharp notch, crack propagation proceeds in a relatively straight line normal to the loading axis (**Supplementary Figure 4a**). By patterning 4 domains in a sample with each oriented at ±45° to the loading axis and the notch, crack propagation can be spatially controlled (**Supplementary Figure 4b**). At the interface of each domain the crack is redirected diagonally towards the soft axis. A crack deflection angle between domains of ~40° is observed. This process is repeated across the width of the sample at the interface of each domain. The crack deflection mechanism can be further utilized to engineer a degree of notch insensitivity as shown in **Supplementary Figure 5**. If a notch is placed in a region of the film where no hard island exists the sample rapidly tears. If by contrast, an elastic domain is placed in the direction of the crack propagation the crack is arrested at the domain boundary. The resulting energy required for failure increases by 400% as compared to a notch that propagates across a single soft domain.
